# Analysis of the microRNA transcriptome and expression of different isomiRs in human peripheral blood mononuclear cells

**DOI:** 10.1186/1756-0500-6-390

**Published:** 2013-09-28

**Authors:** Candida Vaz, Hafiz M Ahmad, Richa Bharti, Priyatama Pandey, Lalit Kumar, Ritu Kulshreshtha, Alok Bhattacharya

**Affiliations:** 1 School of Computational and Integrative Sciences, Jawaharlal Nehru University, New Delhi 110067, India; 2 School of Life Sciences Jawaharlal Nehru University, New Delhi 110067, India; 3 Department of Biochemical Engineering and Biotechnology, Indian Institute of Technology, New Delhi 110016, India; 4 Department of Medical Oncology, Institute Rotary Cancer Hospital, All India Institute of Medical Science, New Delhi, India

**Keywords:** MicroRNA, Next generation sequencing, Normalization, IsomiR, miRnome, Peripheral blood mononuclear cells (PBMC), Chronic myeloid leukemia (CML)

## Abstract

**Background:**

MicroRNAs (miRNAs) have been recognized as one of the key regulatory non-coding RNAs that are involved in a number of basic cellular processes. miRNA expression profiling helps to identify miRNAs that could serve as biomarkers. Next generation sequencing (NGS) platforms provide the most effective way of miRNA profiling, particularly as expression of different isoforms of miRNA (IsomiRs) can be estimated by NGS. Therefore, it is now possible to discern the overall complexity of miRNA populations that participate in gene regulatory networks. It is thus important to consider different isoforms of miRNA as part of total profiling in order to understand all aspects of the biology of miRNAs.

**Results:**

Here next generation sequencing data of small RNAs derived from normal peripheral blood mononuclear cells (PBMC) and Chronic myeloid leukemia (CML) patients has been used to generate miRNA profiles using a computation pipeline which can identify isomiRs that are natural variants of mature miRNAs. IsomiR profiles have been generated for all the 5p and 3p miRNAs (previously known as major mature miRNA and minor or miRNA*) and the data has been presented as a composite total miRNA transcriptome. The results indicated that the most abundant isomiR sequence of about 68% miRNAs, did not match the reference miRNA sequence as entered in the miRBase and that there is a definite pattern in relative concentration of different isomiRs derived from same precursors. Finally, a total of 17 potential novel miRNA sequences were identified suggesting that there are still some new miRNAs yet to be discovered.

**Conclusions:**

Inclusion of different isoforms provides a detailed miRnome of a cell type or tissues. Availability of miRnome will be useful for finding biomarkers of different cell types and disease states. Our results also indicate that the relative expression levels of different isoforms of a miRNA are likely to be dynamic and may change with respect to changes in the cell or differentiation status.

## Background

MicroRNAs (miRNA) are a major class of small non-coding RNAs of about 22 nucleotides that are involved in various cellular functions. miRNAs are transcribed as pri-miRNAs which are processed into pre-miRNAs by an RNase III enzyme, Drosha. Pre-miRNAs are exported to cytoplasm and processed by another RNAse III enzyme, Dicer to give rise to mature miRNAs. These two enzymes are important not only for processing of intermediates to mature miRNAs but also for introducing variations in miRNAs [1-3].

Several reports have established miRNAs as important post transcriptional regulators of gene expression through different mechanisms, such as translational repression and destabilization and cleavage of the target mRNA. The processes are initiated by binding of the miRNA to partially complementary sites at the 3′UTR of the target mRNA in animals and completely complementary sites in plants [4,5]. A single miRNA can bind to several target mRNAs and similarly a multitude of them can bind a single target [6]. Recent estimates suggest that a substantial fraction (up to 60%) of higher eukaryotic mRNAs is regulated by these small non coding RNAs [7]. Therefore, miRNAs and mRNAs are part of intricate networks that regulate gene expression and cellular decision making [8,9]. The existence of miRNA clusters and families also add to the intricacies of miRNA regulation [10]. In order to understand these networks, knowledge of complete miRNA expression profiles is necessary. Expression profiles can also be helpful in identifying tissue, stage and disease-specific patterns. miRNAs have also been shown to be involved in cancer and tumorigenesis [11-13]. It is generally believed that some of the miRNAs can be useful diagnostic and prognostic markers of different cancers with applications in patient care and therapy [14].

A number of approaches, such as northern blotting, RNase protection assay, PCR, microarrays and RAKE assay have been used for expression profiling of miRNAs [15-20]. In general these methods are either cumbersome for scaling up and/or not sensitive enough to detect low levels. miRNAs are expressed at different levels, spanning more than five orders of magnitude and majority of methods are not suitable to get accurate expression profiles. Microarrays have problems associated with cross hybridization and creating a single hybridization condition suitable for all miRNAs [20]. Most of these methods could detect only pre-miRNAs owing to the technical difficulty caused by short length of mature-miRNAs. Next generation sequencing (NGS) has provided an edge over other methods in generating, not just an in depth knowledge of known miRNA expression, but also helped in identifying tissue-specific, and rarely expressed miRNAs [21-25]. NGS approach has also been successful in new miRNA discovery leading to exponential increase in miRBase entries in the last few years (Release 19) [26,27]. Moreover, analysis of small RNA (sRNA) sequencing data derived using NGS platforms has helped to identify alternate processing products of biogenesis. It is clear from some of the preliminary analysis that these alternate processing products are biologically relevant and can have functional role probably by binding different Argonaute proteins [24,25,28,29]. The previously known as minor or “miR*” sequences were found to be expressed higher than the major counterparts raising the need for a change in the annotation. Therefore, the annotation of miRNAs as-5p/-3p rather than mature and star is now used by the latest miRBase Release 19.

In this report, a detailed analysis on human PBMC miRnome has been described. Our analysis involves different processing products that include isomiRs of both miR-5p and miR-3p thus forming a composite miRNA transcriptome. We have generated the reference miRNA profile comprising of the miRNAs in miRBase as well as the abundant isomiR profile using our NGS data. On comparing the reference miRNA and the abundant isomiR profiles we found that the abundant isomiRs differ in different cells suggesting that miRNA profiling must include all the variants for developing biological markers in different diseases.

A few potential novel miRNAs have also been detected during our analysis [30]. Our report highlights intricacies in miRNA biogenesis machinery that can be used as a signature of physiological state of cells.

## Results

### Annotation and apportionment

**Figure 1:**
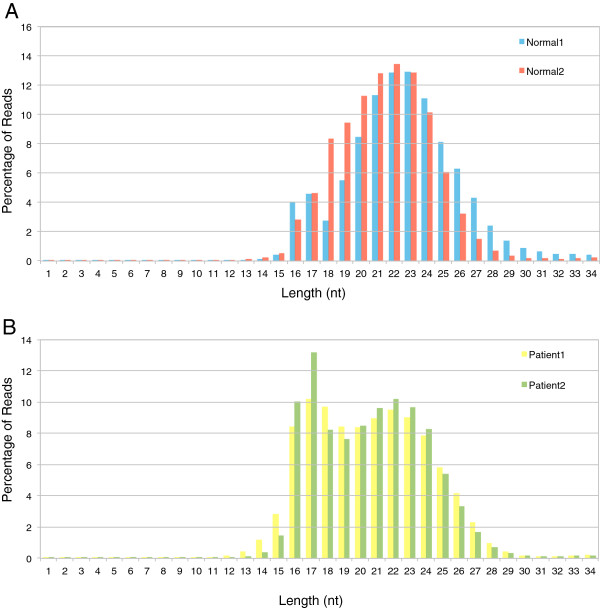
**Analysis of lengths of the reads from the four samples to determine the optimal cut-off read length. A**. Frequency distribution of lengths of the reads from the two Normal samples. **B**. Frequency distribution of lengths of the reads from the two Patient samples.

sRNA datasets comprising of two Normal PBMCs and two CML patients were pre-processed as described in “Methods” and the distribution of the length of the reads was plotted to determine the optimal read length cut-off (Figure 1). Both the Normal samples displayed a normal distribution with the peak at 22nt (average length of mature miRNAs). In contrast, 17nt was found to be the most frequent size of small RNAs in Patient samples indicating higher amount of degradation in these samples. The read length of 14nt was taken as the cut-off length. Therefore reads having lengths greater than 14nt were used for the analysis. The basic statistics about these different datasets is shown in Table 1. The sequences were then analyzed using a computational pipeline that has been described and briefly mentioned in “Methods” in order to classify sequences into different known RNA categories, such as miRNA, sn/sno RNAs, lincRNAs, tRNAs, rRNAs and mRNAs [24]. The reads, that could not be classified (unannotated ones), were further mapped to the intergenic and intronic regions of the human genome and those that matched were extended and used for novel miRNA prediction [30-32].

**Table 1:** Details of the samples

**Sample**	**Total number of reads**	**Total number of reads (> = 14nt)**
Normal1	11,518,605	11,490,402
Normal2	10,878,898	10,855,039
Patient1	10,701,151	10,637,119
Patient2	11,512,769	11,477,587

**Figure 2:**
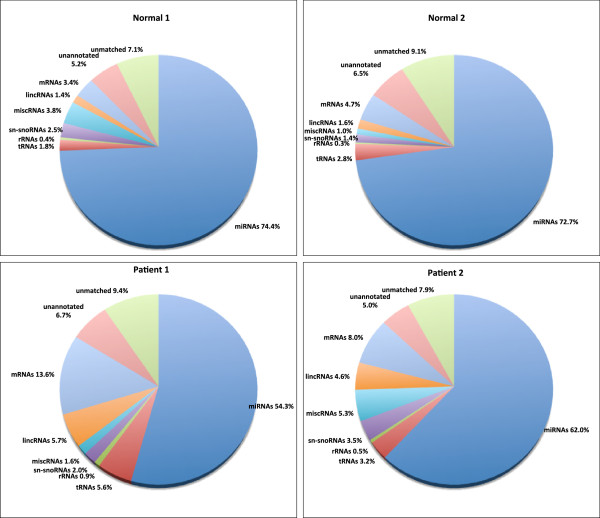
**Distribution of different classes of RNAs present in the four samples.** The pie-charts represent an overview of the small RNAs in the Normal and Patient samples. The miRNAs constitute a major portion of all the four samples; the Normals display a higher proportion of miRNAs as compared to the Patient samples.

Results from the annotation pipeline (Figure 2) showed that miRNAs were the most abundant small RNA species [74% in Normal1, 73% in Normal2, 54% in Patient1 and 62% in Patient2]. Overall the total fraction of miRNAs in the population was relatively less in the Patient samples as compared to that of the Normal samples. The reads derived from other non-coding sRNAs, such as sn/sno RNAs, lincRNAs and miscellaneous RNAs were 1–3%, 1–6% and 1–5% respectively. The reads derived from larger RNA categories, such as the tRNAs were 2–6% and the rRNAs were about 1%. The mRNAs were around 3–5% in the Normals suggesting that degradation was minimal in the Normal samples. However the fraction of mRNAs were slightly higher in Patient samples (8–14%) indicating more degradation in the Patient samples due to cell death and necrosis. This is also evident from the length distribution of the reads (Figure 1).

Reads that matched to the human genome but not with any known RNA species (unannotated reads) were 5–7%. These were taken for novel miRNA prediction. Lastly, the total unmatched pool that failed to match with the human genome was around 7–9% (Figure 2).

### Reference miRNA identification

In general, the stem part of the precursor sequences is processed with Dicer enzyme to give rise to mature miRNAs. Previously, one of the arms was considered to form the mature miRNA whereas the other formed the minor or the star sequence. The expression levels determined the major/minor nomenclature. Recently, several reports have shown that this nomenclature could vary with the tissue and experimental conditions causing the change in the nomenclature. These are now referred to as miR-5p and miR-3p depending on their location in the precursor sequence. The miRBase [26,27] is a repository of miRNAs, and the mature form of miRNAs in miRBase is referred here as the reference miRNAs. The expression profiles of these reference miRNAs were obtained by identifying the reads from our NGS samples that showed an exact match with them.

**Figure 3:**
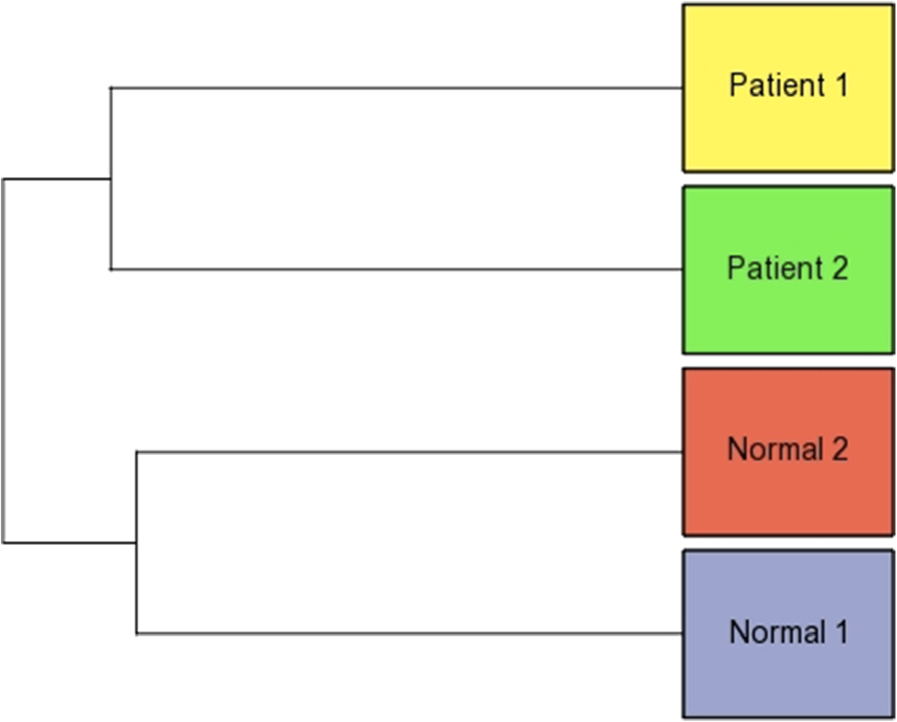
**Hierarchical clustering plot showing the similarity and differences between the samples.** The Normal samples (1 and 2) and the Patient samples (1 and 2) cluster into two separate groups. The plot shows the proximity of the two Normals and the two Patients to each other, and the distance between the Normal group and the Patient group on the whole. Clustering plots were generated using both: the Reference miRNAs expression profile and the Abundant IsomiR expression profile.

The expression profile of these reference miRNAs was called the *Reference miRNA expression profile* (Additional file 1). Furthermore, the similarity and the differences between the samples and within each group, was determined using the Reference miRNA expression profile. The Hierarchical clustering plot computed using the Reference miRNA expression profile, showed that the Normal1 and Normal2 samples were closer to each other forming one group. Similarly, the Patient1 and Patient2 samples were closer to each other forming another group (Figure 3).

### IsomiR identification and examination

miRNAs are also known to have alternative forms called “isomiRs”, that differ from each other by a few nucleotides at the ends. These are thought to be generated by alternate Dicer cutting [23]. Identification of isomiR family has been discussed in the “Methods” section. Briefly, sequencing reads of each sample were aligned to the human pre-miRNAs to obtain an isomiR family for each mature miRNA (-5p/-3p). The Additional file 2 shows an example of a hsa-let-7b isomiR family with their respective frequency or expression values. Quite often only one of these isomiRs displays dominant expression, that is, has highest number of reads among all the other isomiRs. This dominant isomiR is considered to be the active or mature form of miRNAs.

We generated the isomiR expression profiles of all miRNAs and determined the dominant isomiR for each expressed miRNA based on the highest frequency. The Additional file 3 lists the most abundant isomiR for every expressed mature miRNA (*Abundant isomiR expression profile*).

The clustering of the samples was once again checked using the Abundant IsomiR expression profile. The Hierarchical clustering plot computed using the Abundant IsomiR expression profile gave results similar to that obtained using the Reference miRNA expression profile. The Normal1 and Normal2 samples were closer to each other forming one group. Similarly, Patient1 and Patient2 samples were closer to each other forming another group (Figure 3).

We then answered the question whether the most abundant isomiR was the same as the reference miRNA by comparing the Reference and the Abundant isomiR expression profiles. If the most abundant isoform of a miRNA was not the reference miRNA, it was reported as “NO”, whereas if the abundant one matched the reference miRNA it was reported as “YES”. The Additional file 4 lists this comparison.

Analysis of the “YES” or “NO” data revealed some interesting facts (Table 2). We focused on a total of 608 miRNAs that were expressed in both the Normal samples and displayed uniformity in being either YES/NO among them. For the rest of 1434 miRNAs in Normals either the expression levels were low, below the detection limit (1289) or were non uniform (145). Out of 608 miRNAs analyzed, the reference miRNA sequence of only 204 miRNAs matched with the most abundant isomiR (YES cases), while for 404 miRNAs, the reference sequence did not match the most abundant isomiR (NO cases) (Table 2).

**Table 2:** Comparison of the reference and abundant IsomiR expression profiles

**Comparison details**	**Normal samples**	**Patient samples**
**Reference miRNAs that matched the most abundant isomiRs (YES cases)**	204	190
**Reference miRNAs that did not match most abundant isomiRs (NO cases)**	404	429
**MiRNAs not considered**	1434	1423

Comparison of the two expression profiles in Normal and Patient samples.

The same was seen for the patients; around 619 miRNAs were expressed and displayed uniformity in being YES/NO among both the Patient samples. The remaining 1423 miRNAs had either a low expression (1293) or were non uniform (130). Out of the 619 miRNAs analysed, the reference miRNA sequences of only 190 miRNAs matched with the most abundant isomiR (YES cases), while for 429 miRNAs, the reference sequence did not match the most abundant isomiR (NO cases) (Table 2).

The latter category (NO category) included some of the miRNAs that are known to be involved in cancer biology, such as let-7b-3p, let-7d-5p, let-7 g-3p, 10a-5p, 15b-5p, 16-1-3p, 16-2-3p, 21-5p, 21-3p, 23a-3p, 23b-3p, 26b-5p, 26b-3p, 30d-5p. A few examples of such miRNAs for the Normals and Patients are shown in Table 3A,B respectively. Therefore a mature miRNA is not always the same sequence or isoform and appears to vary though the precursor sequence remains the same.

**Table 3:** Few examples revealing differences in the expression profiles based on selection of the most abundant isomiR versus the reference miRNA

**3A)**				
**miRNAs**	**Normal 1**		**Normal 2**	
	**Reference miRNA frequency**	**Most abundant isomiR frequency**	**Reference miRNA frequency**	**Most abundant isomiR frequency**
**hsa-miR-10a-5p**	171	935	195	792
**hsa-miR-15b-5p**	2266	4355	992	2547
**hsa-miR-21-5p**	30955	49979	33091	50565
**hsa-miR-23a-3p**	11160	21009	8375	17718
**hsa-miR-26b-5p**	3409	28934	3685	23018
**hsa-miR-30d-5p**	1449	14156	1893	15233
**3B)**				
**miRNAs**	**Patient 1**		**Patient 2**	
	**Reference miRNA frequency**	**Most abundant isomiR frequency**	**Reference miRNA frequency**	**Most abundant isomiR frequency**
**hsa-miR-10a-5p**	56	256	99	396
**hsa-miR-15b-5p**	1835	2342	1187	4114
**hsa-miR-21-5p**	8868	18032	36712	75907
**hsa-miR-23a-3p**	5793	11774	8296	16159
**hsa-miR-26b-5p**	2239	12346	10093	51801
**hsa-miR-30d-5p**	1638	8012	1619	15705

A) in Normal samples. (B) in Patient samples.

### Expression profile comparison

#### Reference miRNA expression profile

**Figure 4:**
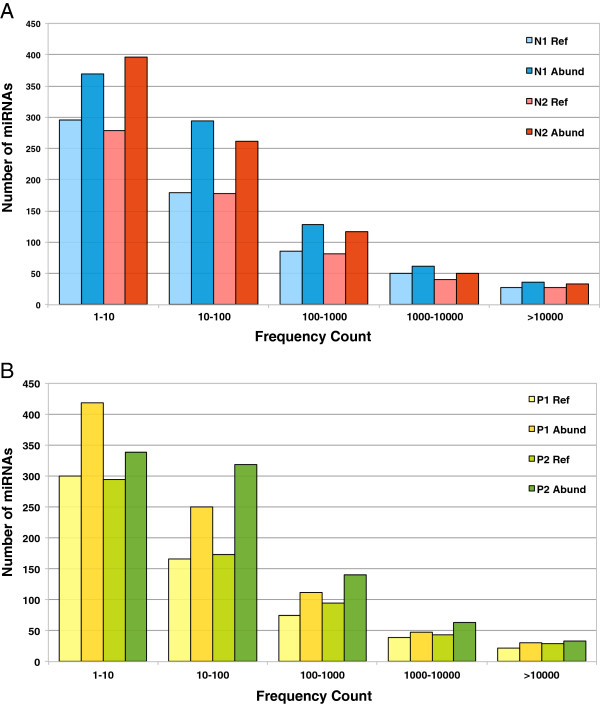
**Analysis of the expression patterns of the reference miRNAs and the most abundant IsomiRs. A**. The distribution of the reference (Ref) and the most abundant (Abund) miRNA levels with respect to number of miRNAs for Normal 1 and 2. **B**. The distribution of the reference (Ref) and the most abundant (Abund) miRNA levels with respect to number of miRNAs for Patient 1 and 2. Numbers of sequence reads are taken as miRNA levels and the values are represented in the form of range of values. The expression levels of the miRNAs span up to five orders of magnitude.

Expression of 620 miRNAs (on an average) including about 70–90 singletons were observed in each sample. The level of expression of the miRNAs was found to be variable (Additional file 1) showing a wide range of values spanning five orders of magnitude in Normals (Figure 4A) and Patients (Figure 4B). The highly expressed miRNAs (>10,000 frequency count) detected by using this profile were the let-7 family followed by hsa-miR-103a-3p, 148a-3p, 16-5p, 185-5p, 191-5p, 192-5p, 223-3p, 24-3p, 25-3p, 26a-5p, 29a-3p, 3184-3p, 320a, 423-5p and 92a-3p similar to what has been seen before [24].

#### Abundant IsomiR expression profile

On an average about 875 miRNAs including about 20–55 singletons were expressed in each sample. Around 255 more miRNAs were found using this abundant isomiR profile that was missed out in the Reference miRNA profile owing to the absence of the reference sequence for these miRNAs.

The level of expression of the miRNAs was found to be variable just like the reference miRNA (Additional file 3) profiles but of a higher level owing to more number of miRNAs detected by using this profile in Normals (Figure 4A) and Patients (Figure 4B). On using this profile we got a bigger list of highly expressed miRNAs (>10,000 frequency count). This included miRNAs found to be highly expressed using the reference miRNA profile as well as comprised of a few more miRNAs that failed to be picked out as highly expressed on using the reference miRNA profile owing to the presence of another isomiR sequence that had a higher frequency than the reference sequence. Some of these miRNAs were: hsa-miR-107, 140-3p, 21-5p, 23a-3p, 26b-5p, 3074-5p, 378a-3p.

**Figure 5:**
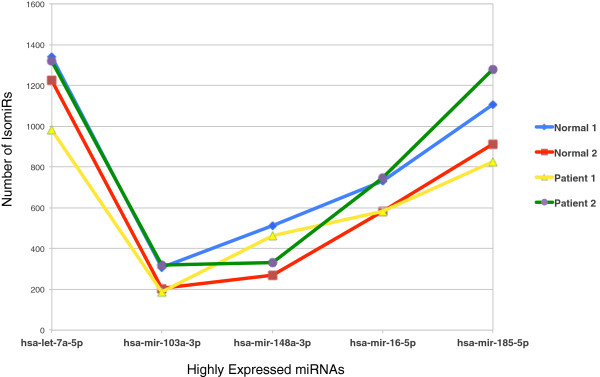
**Distribution of isomiRs in a group of highly expressed miRNAs.** The selected highly expressed miRNAs are hsa-let-7a-5p, hsa-mir-103a-3p, hsa-mir-148a-3p, hsa-mir-16-5p, hsa-mir-185-5p and the patterns appear to be similar across the four samples.

Interestingly, highly expressed miRNAs display more isomiRs as compared to those miRNAs that are expressed at a lower level. Figure 5 shows the distribution of number of IsomiRs of a few selected highly expressed miRNAs among the four samples. The distribution patterns appeared to be similar among the four samples. Number of isomiRs of hsa-let-7a-5p was found to be highest followed by hsa-mir-185-5p, hsa-mir-16-5p, hsa-mir-148a-3p, hsa-mir-103a-3p (in descending order).

### Novel miRNA identification

**Figure 6:**
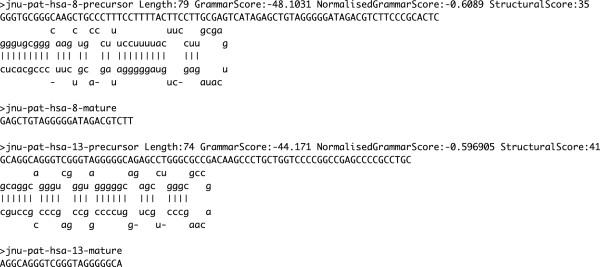
**Sequence and structure of two potential novel miRNAs.** The potential precursors were folded and evaluated by prediction tools, such as CID-miRNA, CSHMM, miRDeep and MiPred. These are two examples of potential novel miRNAs having high frequency or read count.

The reads that did not match with the annotated sequence databases and that mapped to intergenic and intronic regions of the human genome were checked to see if these were potential novel miRNAs. In this case only exact matches were considered for further analysis by extracting the matched region along with 70 nucleotides flanking on either side. The extracted sequences were folded and checked by prediction algorithms, such as CID-miRNA [31], CSHMM [32], miRDeep [33] and MiPred [34] for potential pre-miRNAs. The predictions were further analyzed using a set of filters for false positives as described before [24]. This analysis led to detection of 17 potential novel miRNAs which have been given unique names with the prefix “jnu-pat-hsa”. The sequence and the structures of two potential novel miRNAs is shown in Figure 6. The details of all the 17 potential novel miRNAs, including their frequencies, isomiRs, prediction scores from the tools, the sequences of precursor and mature forms are given in Additional file 5. The structures of the precursors are given in Additional file 6.

## Discussion and conclusions

The major goal of this study was to understand the complete miRNA transcriptome in the context of human PBMCs obtained using deep sequencing of small RNA through custom designed computational tools. The expression of the reference mature miRNAs is commonly considered to form the miRNA transcriptome. Since NGS allows identification of isomiRs of miR-5p and miR-3p, it is important to include these processing variants as part of the complete miRNA transcriptome [23,27,28]. In our previous study we had profiled miRNAs in Normal PBMC and in CML cell line K562 and identified a number of differentially expressed miRNAs [24]. The study did not include human patient cells and isomiR profiling.

The presence of isomiRs was first reported by Morin et.al. 2008 based on their analysis of miRNA sequences obtained through deep sequencing of human embryonic stem cells [23]. These miRNA variants or the IsomiRs have variable 5′ and 3′ extensions. The miRNA transcriptome consists of sum total of different isomiR populations of miR-5p and miR-3p. Among different methods of miRNA profiling, sRNA sequencing using NGS platforms is capable of identifying all these variants. Some recent studies have reported tissue specific accumulation of miRNAs and their counterpart sequences. Preferential expression of IsomiRs has also been reported in tissue specific manner [35]. Profiling of miRNAs in different lymphoid cells has been carried out. For example, one study reported a comprehensive profiling of miRNAs and their isomiRs expressed in B-cells from Jewish centenarians [36]. There is a manually curated database of extracellular circulating miRNAs called the miRandola which is connected to the miRNA knowledge base, making it useful for inferring the potential biological functions of circulating miRNAs [37].

We have used this extended definition of miRNA transcriptome to study the total miRNA transcriptome of Normal PBMCs and CML patients. Since this was a pilot study comprising of two Normal PBMC and two CML patient samples, no attempt was made to identify differentially expressed miRNA isoforms among the normal and patient samples. In general, distribution of miRNA isoforms in normal and patient tissues displayed similar patterns (Additional files 1, 2, 3, 4).

Our results show that mature miRNAs have to be defined with respect to a specific cell type, as relative levels of different isoforms are dynamic in nature. Therefore, we have taken into consideration different isomiRs and looked for the most abundant isomiR and compared with the reference miRNAs (as in miRBase). Our results show that the most abundant isomiR is not always the same as the reference miRNA sequence (submitted to miRBase). This could be due to various reasons, such as tissue specific expression of a specific isomiR or variable degradation rates of different isomiRs. Overall the results presented here suggest that strand selection and processing of miRNAs are likely to be regulated and may be related to phenotypic differences of tissues and cells.

This dynamic nature of isomiRs and their tendency of changing with different conditions can affect any analysis that relies on miRNA expression profile. The Reference miRNA expression profile may not provide an exact representation of the miRNA transcriptome. As our results showed that the Reference miRNA expression profile missed out nearly 255 miRNAs and also identified a fewer number of highly expressed miRNAs, this could lead to incomplete and sometimes a misleading analysis. The Abundant isomiR profile can provide a more appropriate and clearer picture of the miRNA transcriptome and should be used for further downstream analysis such as differential expression estimation and even for finding commonly expressed miRNAs among samples.

Our results on identification of novel miRNAs from sRNA sequences suggest that there are still many novel miRNAs that have not yet been identified. Therefore it is important to find these novel miRNAs in order to define the miRNA transcriptome.

In summary NGS based analysis of sRNA sequences allows complete deciphering of miRNA transcriptome that includes all the isoforms. We are still not clear about the mechanism by which a given cell or tissue decides the functional isoform of a given miRNA and regulation of isoform switching.

## Methods

### Cell line and blood samples preparation

Buffy coat of healthy blood donors (Normal1 and Normal2) were collected from volunteers. Red cell lysis buffer (0.144 M NH4Cl, 0.01 M NH4HCO3) was added to buffy coat to lyse the remnant RBCs and pure WBC population was obtained by centrifugation at 3000 g. Peripheral blood mononuclear cells were obtained at diagnosis from patients with CML after signed informed consent had been obtained from the patient in accordance with the Declaration of Helsinki. This study was approved by the hospital (AIIMS, New Delhi) according to the guidelines of the hospital’s ethics committee (Reference no. A-36:20/10/04).

### RNA isolation and sequencing

Total RNA isolation was carried out from peripheral blood using TRIzol® Reagent (Invitrogen) as per manufacturer’s instruction. RNA preparations were stored at - 80°C till further use. Small RNA population was isolated by separating 10 μg of total RNA on denaturing polyacrylamide gel electrophoresis (PAGE) and cutting a portion of the gel corresponding to the size 18–30 nucleotides based standard oligonucleotide markers. Adapter (5′) was ligated to sRNA population and ligated RNAs (40–60 nt) were purified by running on urea PAGE. This was followed by 3′ adapter ligation and purification of adapter ligated RNAs (70–90 nt) in a similar manner. Modified sRNAs were reverse transcribed and then PCR amplified with adapter specific primers and the amplified cDNAs were finally purified on Urea PAGE to generate cDNA tag libraries for sequencing by illumina genome analyzer.

### Data sets information

The sRNA sequencing data containing PBMC of two normal individuals (Normal1, Normal2) and two CML patients (Patient1, Patient2) were obtained from Illumina high throughput sequencing platform. The sequences shorter than the cut-off read length (14nt), as determined by the read length distribution plot (Figure 1), were removed from all the four samples. The total number of reads for all the samples before and after the application of the read length cut-off is mentioned in Table 1.

### Annotation and data classification

The sRNA sequences obtained were annotated against the known databases using the Elimination pipeline as used in our previous work [24,30]. The Elimination module was used for fast matching of the sequences with the databases. A mismatch of up to 2 nucleotides was allowed. The pool of unannotated sequences at the end of the pipeline served as a source of potential novel miRNAs.

### Reference miRNA expression profile generation

To generate the expression profile of the reference miRNAs, the sRNA sequences of all the samples were matched against the known mature miRNA sequences in miRBase using BLASTN. The parameters used for BLAST were tuned to obtain maximum matches, such as the word size was set to 7 nucleotides, filtering was turned off and the number of alignments reported were increased. The profile comprises of a list of the “reference” miRNAs along with their frequency or expression value for all the 4 samples (Additional file 1).

### Alignment of the reads and isomiR identification

As mentioned in the introduction section, isomiRs are variant forms of the miRNAs caused by alternative Dicer cutting [23]. To obtain the isomiRs for every known miRNA, an alignment of the reads to the pre-miRNA hairpins is necessary. This alignment facilitates identification of isomiRs of mature miRNA sequences derived from both, the 5′ and the 3′ region of the pre-miRNA hairpin.

#### Alignment of the reads to the pre-miRNAs

The alignment was done by the Bowtie software using the default parameters [38]. The pre-miRNAs were downloaded from miRBase Release 19 comprising of 1600 pre-miRNAs and 2042 mature miRNA sequences [26]. Reads from each of the four samples were aligned to the reference pre-miRNAs. The output was an alignment of the deep sequencing reads to the *Homo sapiens* reference pre-miRNAs.

#### Obtaining clusters of the isomiRs

The alignment output was processed by a perl script developed specifically to get a cluster of isomiRs that are actually reads that match at the same location but differ by a few nucleotides at the 5′ and 3′ ends. Such clusters were obtained for every miRNA from both the regions (5′ and 3′ region) of the hairpin using the position information given in the output of the alignment. To demonstrate this, the isomiR family of hsa-let-7b is shown in Additional file 2.

### Abundant isomiR expression profile generation

The isomiR clusters generated as mentioned earlier were analyzed to obtain the most abundant member. An abundant isomiR expression profile comprising of a list of the known miRNAs along with the frequency of the “most abundant isomiR” for all the samples was then created (Additional file 3).

### Comparison of the reference miRNA profile and the abundant isomiR expression profile

The most abundant member could either be the reference miRNA or some other isomiR. Cases where the most abundant isomiR was the reference miRNA itself, was denoted by “YES”, whereas the cases where another isomiR was the abundant one was denoted by “NO” (Additional file 4).

### Novel miRNA prediction

After the NGS datasets were passed through the elimination pipeline to sieve out all the known RNAs, the unannotated ones were matched with the intergenic and intronic regions of the genome to obtain exact matched sequences. Since the intergenic and intronic regions are known to be sources of the miRNA genes, the unannotated RNA sequences were matched for identifying potential novel miRNAs sequences. The exact matches were extended in length corresponding to the average length of a precursor miRNA and then subjected to the ab-initio miRNA prediction algorithms such as the SCFG based CID-miRNA [31] and CSHMM [32] that can predict miRNAs with high sensitivity and specificity.

The ones predicted as miRNAs by these prediction algorithms were further checked for the following features of a miRNA that serve as filters:

1. Presence of the concerned sRNA in one of the arms of the stem region (-5p or-3p) of the hairpin.

2. Presence of one or more isomiR of that sRNA.

3. Presence of another sRNA in the other arm of the stem region (-5p or-3p) of the hairpin.

Following is the detailed pipeline for predicting potential novel miRNAs (Vaz et al., 2010) [24,30]:

i. *Matching and Extending*

The unannotated sRNAs which had frequencies above or equal to 5 were matched to the intergenic/intronic regions. The exact matched sequences were extracted along with 70 nucleotides flanking both the ends to obtain sequences of length comparable to a precursor miRNA sequence.

ii. *Folding and Filtering*

The extended sequences were tested by CID-miRNA [31] and CSHMM [32] prediction tools to find for potential pre-miRNAs. The potential pre-miRNAs reported by these tools were then checked to see if the concerned sRNA occurred in the folded putative precursor and was located in one of the arms of the stem region. Since mature miRNAs are known to be arising from the stem portion and not the loop, only those hairpins in which the sRNAs occurred in the stem were classified as correct cases and the remaining as prediction errors. These correct cases were further tested by MiPred [34].

iii. *Identifying isomiRs and counterparts*

The sRNAs derived from common precursors that were predicted as correct cases, were grouped into a family. The most abundant member of a family was designated as the major mature miRNA. The sRNAs that differed from the representative by a few nucleotides were called its isomiRs and those that had a different, partially complementary sequence and were located in the other strand (stem of the hairpin loop) were treated as its putative counterparts. The Additional file 5 comprise of the potential novel miRNAs grouped into families on the basis of sRNAs falling within the same precursor. The scores from the 4 tools (CID-miRNA, CSHMM, miRDeep, MiPred) assigned to the corresponding precursors are also listed.

iv. *Removing Redundancy*

Finally, the novel miRNA candidates from all the samples were pooled and the redundancy was removed to get a final set of potential novel miRNAs. These were given a unique name and were listed along with their sample IDs. The Additional file 5 comprises the details of all the mature and precursor potential novel miRNAs. The sequence and the structures of two potential novel miRNAs is shown in Figure 6. The Additional file 6 comprises of the structures of all the potential novel precursors.

## Availability of supporting data

The raw data sets supporting the results of this article are available in the Sequence Read Archive (SRA) repository. BioProject ID: PRJNA216976.

BioSample accessions: SAMN02333778, SAMN02333779, SAMN02333780, SAMN02333781.

## Competing interests

The authors declare that they have no competing interests.

## Authors’ contributions

AB conceptualized and along with RK supervised the entire study. LK provided the samples. CV and RB processed and annotated the data using the elimination pipeline. CV did the novel miRNA prediction analysis. CV and PP did the isomiR analysis. RK analyzed the overall data and wrote the discussions and conclusions. HA did the experimental work. CV, RB, RK and AB drafted the manuscript. CV, HA, PP and AB did the revisions. All the authors read and approved the final manuscript.

## Supplementary Material

Additional file 1 **Reference miRNA Expression Profile.** A list of the reference miRNAs, with their raw frequency or expression values as well as the normalized expression values through TPM for all the four samples. TPM (transcripts parts per million) = miRNA Frequency / Total number of reads in the sample * 10^6^. Click here for file

Additional file 2 **An example of a Standard isomiR expression profile.** A list of all the let-7b isomiRs for each sample with position and strand information. Click here for file

Additional file 3 **Abundant isomiR Expression Profile.** A list of the most abundant isomiR, with their raw frequency or expression values as well as the normalized expression values through TPM for all the four samples. Click here for file

Additional file 4 **Classification of the abundant isomiRs as the reference miRNA or not.** Cases where the most abundant isomiR was the reference miRNA itself, was denoted by “YES”, whereas the cases where another isomiR was the most abundant one was denoted by “NO”. Click here for file

Additional file 5 **Details of the potential novel miRNAs.** A list of the potential novel miRNAs predicted sample wise, with the information on their frequency, isomiRs and prediction scores from CID-miRNA, CSHMM, miRDeep, MiPred (Sheet 1). The precursor and mature sequences of the total potential novel miRNAs are given as text file (Sheet 2). All these 17 potential novel miRNAs names have a prefix: “jnu-pat-hsa” and are numbered from 1 to 17. Click here for file

Additional file 6 **Structures of the potential novel miRNAs.** Structures of all the 17 potential novel miRNAs from CID-miRNA prediction tool. Click here for file
