# Supplementary material for: Analysis of the microRNA transcriptome and expression of different isomiRs in human peripheral blood mononuclear cells
Source: BMC Res Notes. 2013 Sep 28;6:390. doi: 10.1186/1756-0500-6-390 (PMC3851811; doi:10.1186/1756-0500-6-390)
Supplement: Additional file 6 — Structures of the potential novel miRNAs. Structures of all the 17 potential novel miRNAs from CID-miRNA prediction tool. [file 1756-0500-6-390-S6.pdf]

```
>jnu-pat-hsa-1-precursor Length:86 GrammarScore:-41.3427 NormalisedGrammarScore:-0.480729 StructuralScore:80
ATTGTGACCCAATATTTACAGGCCCAACATGATCCATATCCCCAGGGAAATGAATCATGTTGGGCCTGTAAATGTTGGGTCACAAT
                                 c   a   c
auugugacccaauauuuacaggcccaacaugau cau ucc c
||||||||||||||||||||||||||||||||| ||| |||
uaacacuggguuguaaauguccggguuguacua gua agg a
                                 a   a   g

>jnu-pat-hsa-2-precursor Length:63 GrammarScore:-37.6935 NormalisedGrammarScore:-0.598309 StructuralScore:18
GAGAACTCCTGGTGCTGAGATGGACGGATCATGATTTCCTTCTGTCTGCACCAGGCTTTGCTT
   aacu-        ug       c   uca
gag     ccuggugc  agaugga gga   u
|||     ||||||||  ||||||| |||
uuc     ggaccacg  ucugucu ccu   g
   guuuc        --       u   uua


>jnu-pat-hsa-3-precursor Length:66 GrammarScore:-40.5417 NormalisedGrammarScore:-0.614269 StructuralScore:16
CTGAGAGGTCAGAAGGGTCAGAATACTGAGGGTCAGAGGCCCTCAGTATTCTCCTTTCCAAGACAG
cugagag   a--      uc              c
       guc   gaaggg  agaauacugagggu a
       |||   ||||||  |||||||||||||| g
       cag   cuuucc  ucuuaugacucccg a
ga-----   aac      --              g


>jnu-pat-hsa-4-precursor Length:66 GrammarScore:-39.6059 NormalisedGrammarScore:-0.60009 StructuralScore:17
GGGAGCGGACGCGGGACGCGGGCGGGGAAGTACCCCGCTGAGCGCGACCTGCCCGGACACGCTCCC
       gac----     a    ---      ga
gggagcg       gcggg cgcg   ggcggg  a
|||||||       ||||| ||||   ||||||  g
cccucgc       cgucc gcgc   ucgccc  u
       acaggcc     a    gag      ca

>jnu-pat-hsa-5-precursor Length:62 GrammarScore:-38.7949 NormalisedGrammarScore:-0.625724 StructuralScore:20
CTGCTTCTAATGCGCAGGCTCGGCCTCCGCCATATTAAAGGTAGCTGGAGCCTTAAGAGCGG
     uc  augcgc      -   cu  gcca
cugcu  ua      aggcuc ggc  cc    u
|||||  ||      |||||| |||  ||    a
ggcga  au      uccgag ucg  gg    u
     ga  ------      g   au  aaau

>jnu-pat-hsa-6-precursor Length:68 GrammarScore:-41.3804 NormalisedGrammarScore:-0.608536 StructuralScore:40
CTGAGTCACGGAGAGGGCGGGAGGCTGCGCCCGTCTGCCCCCGCCACGTCCTCCTGTGAGGGGCTCAG
      ----     a       -  a  cu   cc
cugagu    cacgg gagggcg gg gg  gcg  c
||||||    ||||| ||||||| || ||  |||
gacucg    guguc cuccugc cc cc  cgu  g
      ggga     -       a  g  cc   cu

>jnu-pat-hsa-7-precursor Length:64 GrammarScore:-38.8389 NormalisedGrammarScore:-0.606859 StructuralScore:50
GGAAGCAGGTGGAAGAAAGTGGGCTAAGCTGGCTTGCTGCGCCTTCTGGCTTTCACCTTCCTCC
   a c         aa  u    ua   ug
gga g agguggaag  ag gggc  agc  g
||| | |||||||||  || ||||  |||
ccu c uccacuuuc  uc uccg  ucg  c
   c u         gg  u    cg   uu

>jnu-pat-hsa-8-precursor Length:79 GrammarScore:-48.1031 NormalisedGrammarScore:-0.6089 StructuralScore:35
GGGTGCGGGCAAGCTGCCCTTTCCTTTTACTTCCTTGCGAGTCATAGAGCTGTAGGGGGATAGACGTCTTCCCGCACTC
         c   c  cc  u         uuc   gcga
gggugcggg aag ug  cu uccuuuuac   cuu    g
||||||||| ||| ||  || |||||||||   |||
cucacgccc uuc gc  ga agggggaug   gag    u
         -   u  a-  u         uc-   auac

>jnu-pat-hsa-9-precursor Length:61 GrammarScore:-35.784 NormalisedGrammarScore:-0.586624 StructuralScore:42
GCTCTGGATGGCAGCTGGGAACGTCTTCTTTTAATGAGGGCTCCCCTCTTCCATCCACAGT
   c       c  cu    ac      uu
gcu uggaugg ag  ggga  gucuuc  u
||| ||||||| ||  ||||  ||||||  u
uga accuacc uc  cccu  cgggag  a
   c       u  uc    --      ua
```

```
>jnu-pat-hsa-10-precursor Length:87 GrammarScore:-51.4877 NormalisedGrammarScore:-0.591813 StructuralScore:61
GGGAGGTGAGATGGCGCCTCCTCTCCTTCCTGACACTTTCACTCTGTCGGTGTCTGTGGGAGGTGAGACGACGCCTCCTCTCCTTCC
      u   au    c uc   u      u--      uu a
gggagg gag  ggcg c  cuc ccuucc   gacacu  c c
|||||| |||  |||| |  ||| ||||||   ||||||  | u
ccuucc cuc  ccgc g  gag ggaggg   cugugg  g c
      u   cu    a ca   u      ugu      cu u

>jnu-pat-hsa-11-precursor Length:87 GrammarScore:-50.211 NormalisedGrammarScore:-0.577137 StructuralScore:51
GGGAGGTGAGATGGTGCCTCCTCTCCTTCCTGACACGTTCACTCTCTTGCTGTCTGTGGGAGGTGAGATGGCGCCTCCTCTCCTTCC
      u   au      uc   u      u--    c  ucac
gggagg gag  ggugcc  cuc ccuucc   gaca gu    u
|||||| |||  ||||||  ||| ||||||   |||| ||
ccuucc cuc  ccgcgg  gag ggaggg   cugu cg    c
      u   cu      ua   u      ugu    -  uucu

>jnu-pat-hsa-12-precursor Length:75 GrammarScore:-42.4012 NormalisedGrammarScore:-0.565349 StructuralScore:26
TCTACCATGTTCTGGGCACTGGAACAGTGCCTGGAACTGTTGCTGGAACTGTTACAGTGCCTGGAACTTCTTAGA
    ccau    ug       g      gc ug aac
ucua    guuc  ggcacug aacagu  c  g   u
||||    ||||  ||||||| ||||||  |  |
agau    caag  ccgugac uuguca  g  c   g
    ucuu    gu       a      a- gu guu

>jnu-pat-hsa-13-precursor Length:74 GrammarScore:-44.171 NormalisedGrammarScore:-0.596905 StructuralScore:41
GCAGGCAGGGTCGGGTAGGGGGCAGAGCCTGGGCGCCGACAAGCCCTGCTGGTCCCCGGCCGAGCCCCGCCTGC
      a    cg   a      ag   cu    gcc
gcaggc gggu  ggu gggggc  agc  gggc   g
|||||| ||||  ||| ||||||  |||  ||||
cguccg cccg  ccg ccccug  ucg  cccg   a
      c    ag   g      g-   u-    aac

>jnu-pat-hsa-14-precursor Length:63 GrammarScore:-36.2245 NormalisedGrammarScore:-0.574992 StructuralScore:46
AGGCAGGAAGAGACGCAGCAGCTTGTCTTTGATTCTGCCAAGCTTCTGCATCTTTTCTTCCCT
   c         c    c      u uuu
agg aggaagaga gcag agcuug c   g
||| ||||||||| |||| |||||| |   a
ucc uucuuuucu cguc ucgaac g   u
   c         a    u      c ucu

>jnu-pat-hsa-15-precursor Length:72 GrammarScore:-42.578 NormalisedGrammarScore:-0.591361 StructuralScore:42
CCCTTTCCTTCCATCTCCATCACCTTGAGCATCTTCTGGGCAGCTGAGGTGACCGCAGATGGGAAGGAAGGG
c           -    - ca       g   a--  u
 ccuuuccuucc aucu c  ucaccuu agc   uc u
 ||||||||||| |||| |  ||||||| |||   ||
 gggaaggaagg uaga g  aguggag ucg   gg c
-           g    c cc       -   acg  u

>jnu-pat-hsa-16-precursor Length:72 GrammarScore:-40.4238 NormalisedGrammarScore:-0.561442 StructuralScore:34
TACTGCCTTAACTCTTAGAATCCCCAAAGCATTCTGTGAAGTGGTTTGGGGATTCTAAGAGGAAGAAGAGTA
uac   -   aa--                g    cu
   ugc cuu    cucuuagaauccccaaa cauu  g
   ||| |||    ||||||||||||||||| ||||
   aug gaa    gagaaucuuagggguuu guga  u
---   a   gaag                g    ag

>jnu-pat-hsa-17-precursor Length:100 GrammarScore:-62.2573 NormalisedGrammarScore:-0.622573 StructuralScore:61
GGAGGGCCGCCAACCCGGTCTCCAGTCCTCCCAGTCCCGCTCGGCAGACCCTCGGTGCGCTCGGGGAGACTGTCACGGGAGAAAGGGAAGGTGGCACTCC
    g      aa   gg     a  ccucc     -  g  c   ag
ggag gccgcc  ccc  ucucc gu     caguc cc cu ggc  accc
|||| ||||||  |||  ||||| ||     ||||| || || |||  |||u
ccuc cggugg  ggg  agagg ca     gucag gg gg ucg  uggc
    a      aa   aa     g  cu---     a  -  c   cg
```
